# Modelling the experimental electron density: only the synergy of various approaches can tackle the new challenges

**DOI:** 10.1107/S2052252515007538

**Published:** 2015-05-14

**Authors:** Piero Macchi, Jean-Michel Gillet, Francis Taulelle, Javier Campo, Nicolas Claiser, Claude Lecomte

**Affiliations:** a Department of Chemistry and Biochemistry, University of Bern, Freiestrasse 3, CH-3012 Bern, Switzerland; b Laboratoire Structures Propriétés et Modélisation des Solides, UMR 8580, Université Paris Saclay CentraleSupélec, CNRS, Grande Voie des Vignes, 92295 Chatenay-Malabry, France; c Institut Lavoisier de Versailles, Université de Versailles Saint Quentin en Yvelines, 45 Avenue des Etats-Unis, Versailles, 78035, France; d Materials Science Institute of Aragón, CSIC-University of Zaragoza, Zaragoza, 50009, Spain; e Cristallographie, Résonance Magnetique et Modélisations, CRM2, UMR 7036, Institut Jean Barriol, Université de Lorraine, Vandoeuvre-les-Nancy, BP239, F54506, France

**Keywords:** charge density, spin density, momentum density

## Abstract

Electron density is a fundamental quantity that enables understanding of the chemical bonding in a molecule or in a solid and the chemical/physical property of a material. Because electrons have a charge and a spin, two kinds of electron densities are available. Moreover, because electron distribution can be described in momentum or in position space, charge and spin density have two definitions and they can be observed through Bragg (for the position space) or Compton (for the momentum space) diffraction experiments, using X-rays (charge density) or polarized neutrons (spin density). In recent years, we have witnessed many advances in this field, stimulated by the increased power of experimental techniques. However, an accurate modelling is still necessary to determine the desired functions from the acquired data. The improved accuracy of measurements and the possibility to combine information from different experimental techniques require even more flexibility of the models. In this short review, we analyse some of the most important topics that have emerged in the recent literature, especially the most thought-provoking at the recent IUCr general meeting in Montreal.

## Abstract 2

The most recent research in charge spin and momentum density is presented and discussed.

## 1. Introduction   

Electrons are Fermion particles that adher to Pauli’s exclusion principle, and their distribution in position or momentum space represents a fundamental property for chemistry which is at the heart of all reaction processes and molecular functionalities.

Electrons have a charge, a spin, and because they have a velocity and a mass, they possess a momentum. Consequently, the probability of finding any of the electrons of a system at a given position **r** in space implies an electron charge density ρ(**r**) and an electron spin density ρ^σ^(**r**) (= ρ^↑^(**r**) − *ρ* ^↓^(**r**)). On switching from position space to momentum space, the probability that any electron has a given momentum **p** implies a total momentum charge density π(**p**) and a momentum spin density π^σ^(**p**). Whereas ρ(**r**) originates from the electronic wavefunction in position representation, the so-called momentum charge density π(**p**) is related to the wavefunction in momentum representation. The two alternative representations are related by a simple Fourier transform. Therefore, by virtue of the Heisenberg indetermination principle, the most delocalized electrons bring a dominant but very diffuse contribution to, for example, metallic or covalent bonds in position space, while their momentum counterpart exhibits a sharper feature that is much easier to identify and model. A similar statement can be formulated for spin density in position and momentum representations.

Electron distribution encompasses many sciences (chemistry, physics, biology, material science), but as the electron density is better determined through experiments on crystalline solids, crystallography has always played a dominant role. In fact, ρ(**r**) is a quantum mechanical observable, measurable through scattering techniques: X-ray, γ-ray or electron diffraction for the charge part; polarized neutron diffraction for the spin part; Compton scattering for the momentum charge density and magnetic Compton scattering for the momentum spin density. Due to this diversity, in order to coordinate the research dealing with electron distribution, 40 years ago the IUCr set up a special commission on charge, spin and momentum densities.

In order to explain the importance of modelling these observables, let us focus on the electron charge density in position space. In principle, one could directly obtain ρ(**r**) of a crystal by Fourier summation over all Bragg structure factors *F*_*hkl*_, measured in X-ray diffraction experiments
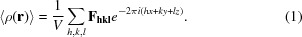
However, this procedure has some practical limitations: (i) the resolution of a diffraction experiment cannot be unlimited and the reconstruction would be biased by the truncation; (ii) while the structure factor modulus is measurable, its phase is not (at least for experiments under kinematic approximation); (iii) the scattering phenomenon actually depends on the thermally averaged electron density, which means the electron density averaged over all possible vibrational eigenmodes of the lattice. In this respect, it is important to remember that nuclei are not steady even at the hypothetical temperature of 0 K.

For these reasons, modelling is a necessity in order to obtain a static electron density distribution, which can reliably represent the quantum mechanical function, obtained with *ab initio* calculations. Some methods, especially those based on the maximum likelihood and Bayesian statistics, reconstruct the thermally averaged electron charge density, a three dimensional function that inherently contains the smearing effect due to atomic vibrations. In principle, this is a more straightforward image of the actual observable. However, for many applications a proper deconvolution of the electron (charge or spin) density from the nuclear probability function is preferable.

Similar arguments would hold true for the determination of electron spin densities. In momentum space, temperature effects are not considered to be much of an issue.

Building a model means parameterizing the electron density distribution in such a way that the measured quantities enable the determination of these parameters. This process is mostly a refinement through non-linear least-squares fitting.

Scientists have adopted the electron density analysis, especially the charge density, for more than five decades, with applications in many fields of chemistry, physics and biology. The accurate modelling of charge distribution became possible only when a significant theoretical background had been developed. This dates back to the early seventies, when many groups understood that the best way to describe the one electron probability density ρ(**r**) was to project it into atomic-like terms with a multipolar shape. Thanks to Kurki-Suonio (1968 ▸), Stewart *et al.* (1975 ▸), Stewart (1976 ▸), Hansen & Coppens (1978 ▸) and Hirshfeld (1977 ▸), this concept found many similar, although not identical, formulations, which allowed for practical applications of charge density analysis. Thereinafter, analyses of electron density maps became very popular, thanks also to the availability of computer programs that could transform models into computable quantities comparable with experimental measures. This has somewhat mirrored the analogous advances made by chemical and biological crystallography in producing software able to rapidly and accurately solve and refine crystal structures.

The multipolar expansion models have further developed, especially for extracting properties directly derivable from the parameterized electron distribution, such as the electrostatic moments, the electric potential, field and field gradients, the electron density derivatives *etc*. Importantly, the multipolar expansion was found useful not only to describe the charge density, but also the spin density (Brown *et al.*, 1979 ▸; Claiser *et al.*, 2005 ▸). In fact, the spin-polarized electron density distribution can also be described in terms of atom-centered multipoles, the coefficients of which are refined against polarized neutron diffraction intensities or flipping ratios (Boucherle *et al.*, 1987 ▸; Ressouche *et al.*, 1993 ▸; Ressouche, 1999 ▸).

Four decades after the first multipolar charge density analyses the field has reached complete maturity, as testified by the large number of research papers published every year in this field, with applications ranging from biology and life science to material science and physics.

The continuous progress of radiation sources and detectors enable the mapping of ever finer features of the electron density distribution. Nowadays, experiments are able to challenge the well established theoretical models and reveal their potential deficiencies (Fischer *et al.*, 2011 ▸), so that new strategies are currently being proposed and systematically tested.

This article will briefly review some of the recent progress, especially that emerging from the recent IUCr meeting in Montreal (hereinafter IUCr2014), focusing on the extension of traditional multipolar models, on the combination of models for charge and spin densities and on the combination of information from theory and experiment.

## 2. More information from modern experiments   

An important issue in charge density analysis has always been the accuracy of the measured data. In fact, because only a small amount of electron density deviates from an ideally spherical distribution around the atoms of the structure, it is extremely important that the scattered intensities be measured with the highest accuracy and precision. Over the years, the improved brilliance of the various sources and the improved quality and rapidity of detectors have contributed to ever more reliable measurements. However, while on one hand there is always room for further progress, on the other hand, good practices should not be abandoned.

In the 1990s, the availability of position-sensitive two-dimensional detectors, charge-couple devices (CCD) or imaging plates (IP) produced a major breakthrough, offering more rapid and complete data sets. Nowadays, the new frontier is that of single photon-counting area detectors that enable rapid read-out, higher dynamic ranges and energy discrimination. Wenger *et al.* (2014 ▸) have recently adapted a pixel area detector on a laboratory diffractometer, showing potential applications for charge density measurements as well as for time-resolved diffraction experiments. An entire micro-symposium was dedicated to this topic at IUCr2014.

In parallel, updated sources for laboratory scale appear periodically on the market, especially after the so-called micro-sources (*i.e.* X-ray sources generated by micro-focused electron beams) have become so widespread. Schulz *et al.* (2009 ▸) and Macchi *et al.* (2011 ▸) have independently analyzed the *pros* and *cons* of these new sources, especially concerning the optics used to focus the X-rays. Undoubted advantages were recognized, although with the warning that contamination of low-energy photons should be carefully checked and eliminated. The new frontier is probably best represented by the liquid metal sources, able to provide an enormous brilliance, but so far only low-energy X-rays are available, unfortunately not sufficient for the specific requirements of charge density studies.

The radiation wavelength of the future is also a matter for debate. For example, Krause *et al.* (2015 ▸) have recently proposed criteria to ascertain for which type of crystals could a high-energy radiation such as Ag *K*α be convenient. In fact, the increased resolution available with a shorter wavelength is undermined by the lower scattering power and lower detector efficiency. However, they could demonstrate a clear benefit for systems containing heavier elements, for which absorption can still be problematic with Mo *K*α. More uncertain instead are the advantages for organic crystals, for which data collections would be very long in order to achieve the requested accuracy.

A chapter on its own is, of course, synchrotron radiation; see, for example, the recent review by Jørgensen *et al.* (2014 ▸). Sources are ever more brilliant and offer a very wide spectrum of energies. Some beamlines at international facilities are committed to providing a highly accurate dataset at a high resolution, as it is necessary for charge density studies as in the study of Sb_3_Co (Stokkebro Schmøkel *et al.*, 2013 ▸). The most relevant methodological outcome in recent years has been the possibility of also obtaining accurate charge density from powder samples; see, for example, Fischer *et al.* (2011 ▸).

The availability of the new technologies would not be sufficient to obtain better results, if good practice and special care were not used during data collection. In this sense, it is remarkable that a number of methods to correct the data, known already in the 1970s, are no longer applied when integrating data measured with modern instruments. In part, this is because the intensity of a given reflection may be collected several times, at different Eulerian angles or on symmetry equivalents. This high redundancy enables the mediation of some common error sources (like beam instabilities) or to empirically correct for them (for example absorption, although a proper analytical correction would always be preferable). On the other hand, repeated measurements are not particularly helpful in tackling other effects, such as thermal diffuse scattering, multiple scattering, sample fluorescence *etc*. At IUCr2014, Sakakura *et al.* (2014 ▸) presented a careful analysis of the effect of multiple scattering on the determination of orbital populations in a series of metal salts. In fact, multiple scattering is also one of those problems that would require careful inspection of the data and that is not normally taken into account by default integration software. Herbst-Irmer (2014 ▸) instead analyzed the effect of data rejection on the quality of a refined model and the problem of over-fitting that could affect multipolar refinements. In this respect, it is important to take into account that software for accurate analysis of massive dataset is missing and the charge density analysis would definitely benefit from such software.

We can conclude that a clear outcome from the recent literature on charge density analysis is that datasets collected using modern technologies undoubtedly contain more information than would be exhausted by models which are too restrictive. For this reason, improvements are being proposed, as summarized in the next few paragraphs.

## 3. More flexible multipolar models for charge density   

The topic of this review article concerns the possibility to extract more information from experimental data, which necessarily means challenging well established models and testing extensions, corrections or even alternative routes. As universally recognized, the ‘standard’ in charge density is the multipolar model, in particular, the formulation proposed by Hansen & Coppens (1978 ▸). Many program packages, developed over the years like *MOLLY* (Hansen & Coppens, 1978 ▸), *MoPro* (Jelsch *et al.*, 2005 ▸), *XD*2006 (Volkov, Macchi *et al.*, 2006 ▸), *JANA* (Petricek *et al.*, 2014 ▸), allow this model to be refined against experimentally measured X-ray diffraction data. Scattering factors measured with radiation different from X-rays (*e.g.* electrons or γ-rays) can also be used, with minor adjustments.

According to the Hansen & Coppens (1978 ▸) model, electron density in a unit cell is first expanded in atomic contribution (as for standard structural refinement)
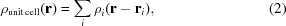
where **r** *_i_* is the position of the nucleus of atom *i*. Each atomic term *i* is further expanded as
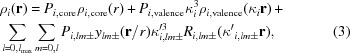
where *P* are population parameters, κ are radial scaling factors, *R*(**r**) are radial density functions, ρ(**r**) are spherically averaged density functions for core and valence, and *y*(**r**/*r*) are spherical harmonics. The indices *l* and *m* run over the angular and azimuthal numbers of spherical harmonic functions, respectively. In the *standard* Hansen and Coppens model, all population parameters of equation (3) are typically refined, but core populations are kept fixed. Symmetry or chemical constraints may be applied, so that the number of refined parameters is actually smaller. In particular, radial scaling parameters of all multipoles with *l* > 0 (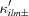) are normally constrained to be the same for a given atom and all atoms of a given element-type share the same set of κ and κ′ and *R*(*r*) functions.

Since the very beginning it was clear that some limitations of the atom-centered multipolar expansion could have undermined the possibility of retrieving the most sophisticated features of electron density. Here we summarize these limitations:

1. (*a*) The ‘two-center electron density’ is not accounted for. In fact, the electron density from the product of the orbitals centered on two atoms is approximated by combinations of one-center functions. Noteworthy is the expansion of electron density in atomic terms which means a more severe approximation compared with the expansion of molecular orbitals as combinations of atomic orbitals (as adopted in most of the quantum chemical calculations). The approximation could affect the precise description of the bonding electron density (Bentley & Stewart, 1973 ▸). While this component is often mildly considered when pure position charge (or spin) densities are the primary point of interest, its importance (as detailed below) can no longer be ignored when momentum space is to be accounted for.
2. (*b*) The angular expansion is truncated. In principle, the electron density expansion would be exact if each center had an infinite number of spherical harmonics, which is obviously not feasible. On the other hand, in order to obtain suitable convergence through least-squares refinement, the expansions are usually limited to the hexadecapolar level for main group and transition elements. The hexadecapolar expansion is strictly mandatory for *d*-block elements, because the product of *d*-orbitals implies a combination of spherical harmonics up to *l* = 4 in equation (3). For *p*-block elements, this is not mandatory. However, the strong two-center character of the electron density in organic molecules requires an expansion well above the simple product of *s* and *p* atomic orbitals (which formally implies only monopole, dipole and quadrupolar functions). Elements of *f*-block would instead require a hexacontatetrapole expansion [*i.e.* up to *l* = 6 in equation (3)], which is however seldom used due to the lack of a sufficient amount of data. *JANA* (Petricek *et al.*, 2014 ▸) and *MoPro* (Jelsch *et al.*, 2005 ▸) enable such high expansion.
3. (*c*) The radial part is poorly described. Only one radial function per each orbital density is used or, at best, a contracted multiple-zeta function. This is a clear limitation, especially for the valence density. In quantum chemical calculations, single zeta basis sets rarely produce sufficiently accurate results and are normally not adopted. Interestingly, this subject was initially discussed exactly in one of the seminal papers on the multipolar model, the famous Hansen & Coppens (1978 ▸) paper, where correct exponents for the valence shell of S atoms were tested. The issue remained quite silent for sometime, until some studies in the late 1990s reopened the debate.
4. (*d*) The core electron density is frozen. In order to minimize the number of parameters, charge density studies typically neglect distortions of core electrons, keeping the population and the radial distribution fixed to that calculated for the atom in isolation. Nevertheless, as anticipated since the 70s (Bentley & Stewart, 1974 ▸), core distortions could occur and become visible with X-ray diffraction experiments if the quality of the data and the resolution collection were improved.
5. (*e*) Position and thermal motion of H atoms are inaccurate. In basic crystallographic courses, it is usually taught that H atoms are invisible under X-rays. This is not exactly true, although it is obvious that their modeling is more problematic. The single electron of H, entirely involved in the chemical bonding (including maybe hydrogen bonding), is obviously very elusive and the large and often anharmonic motion of H increases the ambiguity. Over the years, many models have been proposed to partially solve this problem. Stewart (1969 ▸) in a seminal paper proposed a generalized scattering factor for an atom covalently bonded and he used in fact H as a reference. The method was later included in the program *VALRAY* (Stewart *et al.*, 2000 ▸) and could be used to estimate surprisingly accurate positions of the H atoms based on X-ray data only.

Most of these issues did not concern too much the charge density studies of the 70s, 80s and early 90s, mainly because the data accuracy was not sufficient to reveal model deficiencies. It was only after the introduction of modern detector techniques that some of them emerged and fostered the search of alternative solutions.

While for a proper description of the two-center density, point (*a*)[Other l1li1], alternative models are necessary; all the other issues have been somewhat included in modified versions of the original Hansen & Coppens (1978 ▸) formalism. In fact, angular expansion is in principle unlimited, although practical reasons restrict the model refined to *l* = 4. Easier availability of higher resolution datasets enables such an extension. Many studies have proposed more flexible radial functions for the aspherical terms of equation (3) (Iversen *et al.*, 1997 ▸; Volkov *et al.*, 2001 ▸), although their introduction could be at the expense of the stability of the refinement procedure. In fact, many recipes have been proposed to reduce the flexibility by applying sensible constraining, especially important for the κ parameters (Volkov *et al.*, 2001 ▸). In the past few years, some studies have investigated the deformations of the core electron density. By default this is kept spherical, as in equation (2), but a straightforward modification of the model enables the refinement of a set of multipoles and contraction/expansion parameters for the core as well. The first interesting results concerned diamond and silicon (Fischer *et al.*, 2011 ▸; Bindzus *et al.*, 2014 ▸). Although based on powder diffraction data, the accuracy of the measurements was sufficient to enable detailing the effects of chemical bonding on core electron densities. At IUCr2014, Wahlberg *et al.* (2014 ▸) reported on a similar investigation of the isomorphic BN solid, although refinements on this species are statistically less stable than those on silicon and diamond.

## 4. Charge and spin densities in position representation from combined X-ray and polarized neutron diffraction   

One of the most challenging goals in modeling the electron density is a simultaneous refinement of charge, spin and momentum distributions. Within this framework, many efforts were spent in developing a model able to jointly correlate the experimental information from different sources (X-ray diffraction, polarized neutron diffraction and Compton scattering). At the present stage, an intermediate step has been presented by Claiser *et al.* (2014 ▸) and Deutsch *et al.* (2014 ▸), namely the simultaneous refinement of charge and spin density distribution, obtained by refining the parameters of a multipolar model against X-ray and polarized neutron diffraction (PND) data.

While XRD and non-polarized neutron diffraction data consist of integrated intensities of Bragg reflections, PND measures ‘flipping ratios’ [hereafter denoted 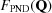]. They are defined as the ratio between the diffracted intensities for spin up and spin down incident neutrons. PND gives access to magnetization density that is the sum of pure spin density and orbital contribution (Schweizer, 2006 ▸). From the above considerations, it appears quite clearly that XRD and PND consider electron distribution from different and complementary perspectives. XRD enables the reconstruction of total electron distribution, ρ(**r**), while PND provides information which yield the spin density, ρ^σ^(**r**)
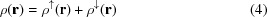


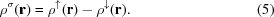
It is therefore obvious that a combined analysis of accurate high-resolution X-ray and polarized neutron diffraction data should yield unprecedented access to spin-resolved electron densities for crystals with significant magnetic properties.

In order to achieve such a joint analysis, a ‘spin-split pseudo-atoms model’ was adopted, derived from the above mentioned Hansen–Coppens model (Deutsch *et al.*, 2012 ▸). ρ(**r**) then writes
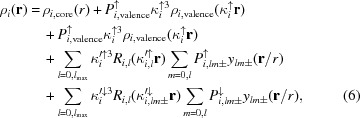
where 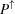 and 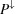 refer to spin up and spin down parameters, respectively. Thus, the challenge consisted of the determination of 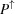 and 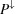, as well as 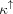 and 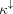, against XRD and PND data in a unique refinement procedure with an appropriate weighting scheme. The method has been successfully tested on a dicopper complex in which the Cu^2+^ ions are coupled by two azido bridges (N_3_^−^) (Aronica *et al.*, 2007 ▸). Spin up and spin down electron multipole density maps have been calculated for the first time, which has made it possible to successfully discriminate the density probability distribution of spin up and spin down electrons. Density functional theory calculations [at the B3LYP/6-31++G(d,p) level] were carried out on an isolated molecule in its experimental geometry. The theoretical and experimental distributions compare extremely well. The spin up distribution in the vicinity of the copper nucleus is spherical, while the down spin distribution shows maxima in the *d_xy_* direction of the ligands. Thus, most of the electron anisotropy around the Cu atom should be attributed to spin down electrons. This is confirmed by a *d*-type function analysis (Holladay *et al.*, 1983 ▸): 30% of spin down electrons lie in the *d_xy_*-type function with corresponding 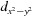 depletion (8%), while all *d_xz_*, *d_yz_* and 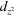 are almost equally populated. One utmost consequence of the spin-resolved model is that it is shown for the first time that the valence spin ↑ density is 5% more contracted than the spin ↓ density [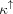 = 0.998 (1), 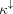 = 0.943 (1); Deutsch *et al.*, 2014 ▸], in agreement with some theoretical predictions. As reported by Claiser *et al.* (2014 ▸) at IUCr 2014, this method has been successfully applied to an organic radical.

## 5. Charge and spin density information from NMR   

Within crystallography, the number of studies based on NMR spectroscopy is increasing. In fact, in structural chemistry and biology, NMR (including solid-state NMR) is the most complementary technique for diffraction methods. Although the electron density community has made only very little use of NMR spectroscopy, normally limited to finding confirmation of atomic charge states, at IUCr2014 combined X-ray and NMR investigations of the structural and magnetic properties of materials have appeared in a dedicated micro-symposium. In the following, we will briefly review the basic concepts of solid-state NMR, highlighting possible source interplay with electron density analysis.

In solid-state NMR, a constant magnetic field polarizes the nuclear magnetic moments and subsequent application of a radio-frequency magnetic field induces transitions between magnetic states of the nuclei. In addition to the splitting of energy levels induced by the constant magnetic field, all the interactions occurring within the structure that could perturb the nuclear magnetic moments affect the energy levels as well. These interactions are inherently anisotropic; therefore, they depend on the relative orientation with respect to the applied magnetic field (the ‘space part’ of the interaction), on the magnetic state of the nuclei, and on the orientation of nuclear magnetic moments with respect to the main magnetic field (the ‘spin part’ part of the interaction). The anisotropic character of the interactions leads to a general decomposition into irreducible spherical tensors. The observed transitions of nuclear magnetic states provide all the information on internal interactions, and therefore about the nuclear position. Solid-state NMR possesses phase coherence and takes advantage of the continuously modulated orientation of the sample in the magnetic field. This leads to the different schemes of sample reorientation, with a special emphasis on magic angle spinning. Sample reorientation selectively averages the various Hamiltonians depending on the symmetry of the pulse sequence. When its spin part evolution is properly aligned, all spin can be coherently manipulated. This is the basis of high-resolution NMR that allows one to ‘edit’ different components of the Hamiltonians using a proper pulse sequence.

The geometry of a crystal (*i.e.* the relative positions of nuclei) becomes visible by analyzing interactions that define the various energy levels. Each interaction implies a given energy; therefore, it is associated with a Hamiltonian operator, and it contains a space and a spin component
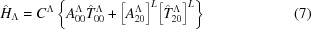


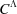
 is a number defining the relation between the dimensionless *A* (space part) and *T* (spin part) terms and the effective energy. Usually, these terms contain geometrical information, *i.e.* distances, angles or connectivity neighbors. The *A*_00_ *T*_00_ term defines the isotropic part of an interaction (when it exists) and the *A*_20_ *T*_20_ term defines the anisotropic part. The latter can be modulated by space or spin manipulation.

Many interactions Λ may be simultaneously active on each nucleus. To mention a few, the shielding produced by electron current (or chemical shift interaction), the indirect nuclear spin coupling mediated by electrons spin coupling, the direct dipolar nuclear spin coupling, the quadrupolar interaction between the nucleus and the electrical field gradient generated by anisotropic charge distribution, the hyperfine interaction between nuclei and electrons, the Knight shift between conduction electron and nuclei. A detailed theoretical description of all these interactions characteristic of the sample would result in an infinite number of terms in equation (7). To overcome these intrinsically unlimited levels of complexity, all the interactions, written as irreducible spherical tensors, can be systematically engineered selecting the physical measurements that will define them, the pulse sequence, and their relation to the geometry of the interactions that are extracted.

Because each interaction forms an extremely complex set of energy levels, their selective editing is the rational way to unravel this complexity. Actually, the adapted choice of the sequence allows, ideally, the suppression of all interactions but the one of interest. The strategy is therefore unique compared with diffraction methods, for which there is no space or time resolution of the diffraction event in the sample (at least with standard techniques). In an NMR investigation, each structural parameter may be associated with a separate signal. Diffraction provides directly high statistics on the set of distances, and separation of different diffracting lines is best achieved with a single crystal by separating each orientation one by one (*i.e.* by rotating the crystal). In NMR, statistics are done in a sense afterwards, because many interactions have the same irreducible spherical tensor for the space part. Thus, geometrical information appears redundant in the different NMR observables edited by different experiments, increasing the reliability of the space part of the interactions (Taulelle, 2009 ▸).

All these interactions carry information on thermally smeared electron density, *i.e.* averaged over all possible vibrational eigenmodes, which is an apparent static electron density. Each interaction can be edited into pieces with the selection of its symmetry by proper selection of combined motion of a sample and its synchronized motion of nuclear and electron spin. Modeling these interactions might become a formidable task, but can be broken into smaller pieces and recombined afterwards into a picture, the accuracy of which could be tuned depending on the needs. Much improvement in modeling has been achieved especially using gauge-included Projector augmented wave computing methods (Bonhomme *et al.*, 2012 ▸).

These measurements can be organized like the pieces of a puzzle. They may give access first of all to several partial distance matrices using the direct dipolar coupling between nuclei of the same (homonuclear) atoms or of heteronuclear nature and different homo- or heteroradial distribution functions. Qualitative connectivity can be edited by indirect couplings, providing topological organization of the crystal. Then electron distribution in their different wavefunctions can be described by all anisotropic interactions of nuclei with electrons. This progressive building of a crystal picture can be mapped onto a picture extracted from diffraction methods. The averaging of methods over space and time is different so the pictures must not coincide. Most diffraction methods would extract the symmetrically periodic part of the crystal structure, while NMR may average in space without periodic filtering. From such differences furthering of the crystal description can be carried out, see Taulelle *et al.* (2013 ▸) and Martineau (2014 ▸).

So far, crystallographers have made limited use of the spin density information available from a NMR measurement, nevertheless the increasing number and quality of experiments will likely offer more opportunities.

## 6. The interplay between position and momentum space   

When attempting to give a thorough description of electron distribution in solids and its influence on the nature of chemical bonds, one should bear in mind that, notwithstanding its obvious connection to our representation of the world, position space is not the only particular representation that is offered for a quantum state. Over the last 40 years, and more specifically since the advent of high brilliance synchrotron radiation sources, inelastic X-ray scattering in the high-energy and momentum transfer regime, *i.e.* X-ray Compton scattering, has become an increasingly popular method to observe electrons from a momentum perspective (Hayashi *et al.*, 2002 ▸; Cooper *et al.*, 2004 ▸).

Since they originate from two different representations of an N-particle wavefunction, electron charge (or spin) densities in position and momentum spaces are not related in a straightforward manner. As it turns out, the shortest path that connects those two quantities has long been established (Coulson, 1960 ▸) to go through the one-electron Reduced Density Matrix (1-RDM)
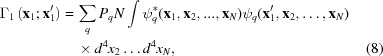
where 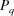 is the temperature-dependent probability for a pure state represented by the *N*-particle wavefunction 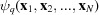 with 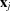 representing both the position 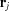 and the spin coordinate of electron *j*. The position charge density is thus obtained by merely setting 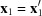, while a particular X-ray Compton scattering spectrum, corresponding to a given direction 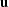 of the scattering vector, yields the so-called Directional Compton Profile 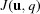. As the latter is nothing but the projection of electron density in momentum space onto the scattering vector, its relationship to the 1-RDM writes (Weyrich, 1996 ▸; Schmider *et al.*, 1992 ▸, 1993 ▸)
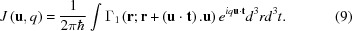
This expression shows quite clearly that Compton scattering observes a different part of the 1-RDM than X-ray diffraction. While the latter gives access to the diagonal part: 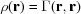, the former offers an indirect measurement of its off-diagonal regions. Therefore, this difference in the probing abilities of each technique also emphasizes their respective roles in our understanding of the wavefunction. On one hand, charge density gives an accurate description of the local behavior, where it takes its largest values, *i.e.* the immediate surroundings of each nucleus. On the other hand, the momentum description highlights the delocalized structures of the wavefunction and the coherent contributions of each site. Of course, there is no clear-cut frontier and, as both quantities address some mean electron behavior, one should expect the combination of these two points of view to bring a mutual reinforcement in the accuracy of each electron density representation.

The power of X-ray diffraction and position space representation of charge density has clearly been stated above. On the other hand, it is well accepted that there are numerous obstacles in interpreting Compton profiles on their own and on an absolute scale: more often than not, for example, differences between profiles have to be performed (Sakurai *et al.*, 2013 ▸). Moreover, it is not easy to think of the chemical bond machinery from a momentum perspective and, to this day, there is no generic model, equivalent to the one brought by Hansen & Coppens, for a momentum density interpretation of Compton scattering data. One is thus often left with no other choice than a simple, but informative, comparison with *ab initio* quantum computations (such as *CRYSTAL*v; Erba & Pisani, 2012 ▸). With the exception of some modest attempts (Gillet, 2007 ▸), it is even more true for a joint interpretation of directional Compton profiles and structure factors in terms of the 1-RDM elements. On many occasions during IUCr2014, there were many discussions and remarks underlining the necessity of considering Compton scattering as a precious additional contribution to a fair description of electronic behavior in molecules and solids. Despite the technical difficulties in making it effective, the community acknowledges that such a joint approach should be further explored, in particular, when delocalized mechanisms are to be evoked, such as in the case of spin magnetism.

## 7. Merging theory and experiments   

In an orthodox interpretation of a science, experimental observations should be as independent and unbiased as possible from the theoretical predictions and *vice versa*. However, in modern crystallography, this entanglement is already quite tight and almost inseparable, even for routine crystal structure determinations. In fact, while chemists normally consider the refined geometries as the result of pure ‘observations’, they do, in fact, contain a large amount of theory: for example, the atomic form factors used for the calculations of structure factors are not ‘observed’, but come from the Dirac–Fock wavefunctions computed for all atoms in isolation (Maslen *et al.*, 1992 ▸). When considering charge density analysis, the influence of theory is even larger because almost all the functions used in equation (3) to describe the electron density models have a theoretical origin. Core and spherical valence terms are typically taken from Roothan’s expansion of atomic orbitals, calculated on isolated atoms at the Hartree–Fock level or, in order to include relativistic effects, at he Dirac–Fock level. In this context it is difficult, therefore, to state that an experimental electron density is truly 100% experimental. Nevertheless, there is a consensus to consider as ‘experimental’ the valence density obtained during a multipolar refinement, given that, in general, the flexibility of a multipolar model is sufficiently high. On the other hand, the core electron density is typically kept frozen, apart from in the recent studies aimed, in fact, at investigating core polarizations.

In the past two decades, some methods have been proposed to even strengthen the connection between experimental measurements and calculations. Among these methods, the X-ray constrained wavefunction proposed by Jayatilaka (1998 ▸), Jayatilaka (2012 ▸) and Jayatilaka & Grimwood (2001 ▸) has received much attention and is still under constant development. The method is based on a modified self-consistent-field approach to obtain a *pseudo*-quantum mechanical wavefunction. Instead of minimizing the expectation value of the Hamiltonian operator, this approach includes a restraint to the residual electron density. Thus, the calculated wavefunction is the one that minimizes the energy under the condition of also minimizing the difference between calculated and measured X-ray structure factors with an appropriate weight. The link is applied through a Lagrangian multiplier, which determines how much the experimental data should be used. While the wavefunction is calculated for an isolated molecule, the link to experimental structure factors implies accounting for crystal field effects as well. Therefore, this procedure introduces a multifaceted perturbation to the molecular wavefunction through the experimental measure; in particular, the effect of a crystal field and the effect of the (exact) electron correlation.

At IUCr2014, Genoni (2014 ▸) reported on new developments of this approach, namely the X-ray constrained extremely localized molecular orbital approach (Genoni, 2013*a* ▸,*b* ▸; Dos Santos *et al.*, 2014 ▸). Following Jayatilaka’s method, the wavefunction is calculated with the additional constraint that molecular orbitals are centered on atoms or bonds, following the scheme proposed by Stoll *et al.* (1980 ▸). The novelty of Genoni’s approach is that the X-ray constrained wavefunction would preserve the chemical interpretability of the multipolar approach, because simple atoms or fragments could be extracted. This is very useful for the portability of the calculated coefficients, a topic that has attracted much interest within the transferable data bank approaches (*see below*).

Another method that is emerging, again combining theoretical calculations and experimental measures, is the Hirshfeld Atom Refinement (HAR), proposed by Jayatilaka & Dittrich (2008 ▸) and by Capelli *et al.* (2014 ▸). HAR is based on the Hirshfeld stockholder partitioning of the electron density (Hirshfeld, 1977 ▸), through which one can define an atom in a molecule and therefore a scattering factor. In the HAR refinement, theoretical calculations provide the aspherical atomic scattering factor, used for refining other parameters. In particular, Capelli *et al.* (2014 ▸) were able to challenge the statement by Hirshfeld (1976 ▸) that atomic thermal motion of H atoms cannot be determined from X-ray diffraction data.

## 8. Applications of the electron density   

From the previous paragraphs, it is clear to the reader that obtaining an accurate electron density distribution is a rather complex, although feasible, task. These efforts would be, however, wasted if significant and useful information were not extracted from the refined models. In fact, the electron density determines a number of properties that reflect the main features of a system, such as atomic charges, electric moments, magnetic moments, bonding electron density, electric forces acting on atoms and molecules *etc*. A comprehensive overview is beyond the scope of the article and the reader is referred to some recent literature (Gatti & Macchi, 2012 ▸; Macchi, 2013 ▸).

In the charge density analysis, the applications mainly concern the analysis of the chemical bonding, especially within the framework of the quantum theory of atoms in molecules (QTAIM; Bader, 1990 ▸), or the determination of electrostatic properties and interactions.

The QTAIM has been quite systematically adopted on experimentally refined models of charge density for the past two decades. A known limitation of the information available from standard multipole models is that some quantities typically used in theoretical QTAIM analyses, such as energy densities and electron delocalization indicators, are not directly available from expansion of equation (3), because they would require knowledge of the whole first-order reduced density matrix and not only its trace (*i.e.* the electron charge density itself). These limitations are somewhat overcome if X-ray constrained wavefunctions are calculated (Genoni, 2014 ▸) or, in principle, if reduced charge density matrix components are directly refined (Gillet, 2007 ▸). Moreover, Abramov (1997 ▸) demonstrated the possibility to approximate the kinetic energy density based only on charge density, its gradient and Laplacian, therefore quantities directly available from standard multipolar models. The Abramov approximation has enabled dissociation energies of hydrogen-bonded (HB) aggregates to be quantified, as originally proposed by Espinosa *et al.* (1998 ▸). Brezgunova *et al.* (2012 ▸) recently used the same approximation for other intermolecular interactions, such as halogen bonding.

More complicated is the possibility of retrieving information on electron delocalization, knowing only multipolar charge density. In an attempt to overcome these limitations, Gatti (2012 ▸) proposed the use of the source function *S*(**r**,**r**′), developed by Bader & Gatti (1998 ▸), which is an influence function (Arfken, 1985 ▸) for the electron density
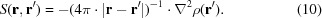
By integrating *S*(**r**,**r**′)*d* **r**, the total electron charge density ρ(**r**) results. Although the source function depends on the charge density and its derivatives only, it is supposed to reflect, at least in part, the electron delocalization occurring in molecules (Gatti, 2012 ▸). This interpretation has actually received some criticism (Farrugia & Macchi, 2009 ▸), although it has been applied in quite a number of experimental studies. At IUCr2014, Gatti *et al.* (2014 ▸) proposed a spin-polarized source function, able therefore to increase the information by defining the influence function for each spin-density component. Although only theoretical examples have been proposed so far, the spin-polarized source function could be straightforwardly calculated from joint charge and spin density multipolar models (Deutsch *et al.*, 2014 ▸) refined against experimental data.

The connection between the topology of charge density and chemical reactivity is another issue that is currently attracting interest, see for example Ayers *et al.* (2015 ▸). The possibility of extracting from charge density suitable indicators not only of the chemical bond strength but also of the chemical reactivity is obviously a long standing issue, and ongoing efforts may finally enable various theories to be unified. Other attempts were made to evaluate non-covalent interaction energy from electron density parameters. This should enable one to estimate the lattice energy of a crystal as the sum of intermolecular interaction energies. Shishkina *et al.* (2013 ▸), for example, showed that the obtained value for the lattice energy was in reasonable agreement with both the experimental sublimation energy and the *ab initio* lattice energy.

The other important outcome of a charge density analysis is the determination of electrostatic properties of atoms and molecules and the evaluation of electrostatic interactions between them, in aggregation. Thanks to Su & Coppens (1992 ▸), Stewart & Craven (1993 ▸), Ghermani *et al.* (1993 ▸, 1992 ▸) and Volkov, King *et al.* (2006 ▸), electric potential and derivatives can be derived from the multipolar expanded electron density. These studies have opened new opportunities for research in this field, in particular, for the recognition of electrophilic and nucleophilic regions in a molecule, packing effects in crystals, docking in proteins (Jelsch *et al.*, 2011 ▸; Muzet *et al.*, 2003 ▸; Li *et al.*, 2002 ▸), surface charges in solids, polarizabilities of molecules and optical properties of crystals *etc*.

The most widespread analyses are based on molecular electrostatic potential, used since the 1980s (Politzer & Truhlar, 1981 ▸; Gadre & Shrivastava, 1991 ▸) to anticipate reactive sites of molecules and packing efficiencies of molecules in crystals. Originally based only on theoretically computed electrostatic potentials, these studies found many applications also using experimental charge densities (see, for example, Bouhmaida *et al.*, 1997 ▸; Fournier *et al.*, 2009 ▸). The analyses of experimentally derived electric potential focused on molecular recognition, especially hydrogen bonding and, more recently, halogen bonding (see Bui *et al.*, 2009 ▸; Pavan *et al.*, 2013 ▸).

In recent years, attention was also concentrated on the first derivative of the electric potential, namely the electric field (EF), see Volkov, King *et al.* (2006 ▸). Being a vector, the EF visualizes the forces and therefore their directionality, giving a more comprehensive picture of the mutual perturbation produced by interacting molecules. Bibila Mayaya Bisseyou *et al.* (2012 ▸) have, for example, computed the total forces acting on atoms in coumarin, by means of an experimental multipole model and a transferable multipole database (Domagała *et al.*, 2011 ▸). At IUCr2014, Guillot *et al.* (2014 ▸) stressed the importance of these results, especially if applied to structural biology. In addition, they demonstrated the importance of visualization tools, useful to better appreciate the information available from the calculated electrostatic field. An interactive tool to explore the electric fields in a crystal seems to be feasible now, following, for example, the analogous system proposed by Haag *et al.* (2014 ▸) to explore the chemical reactivity.

New interpretative tools based on electron density are also emerging that enable the assessment of a broad spectrum of intermolecular interactions, not only those based on electrostatic forces. In particular, reduced density gradients (RDG) and the corresponding non-covalent interaction plots have attracted much attention (Johnson *et al.*, 2010 ▸). Like the source function, reduced density gradient analysis is also based on charge density and its derivative only; in fact
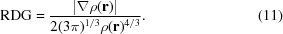
The easy formulation of RDG implies that *ab initio* calculated or multipolar refined electron densities are interchangeable (Saleh *et al.*, 2013 ▸). Attempts to extract information on the actual energy associated with the RDG features have recently been proposed by Saleh *et al.* (2015 ▸). They used approximated energy density functions (Abramov, 1997 ▸) which provide some correlations with characteristic NCI plots. This area is still quite unexplored and applications will certainly be tested in the near future.

## 9. Conclusions and outlook   

This review article focused on the potential of electron density analysis in view of the latest advances. In particular, we showed that the various synergies currently available, mixing different experimental techniques or experiment and theory, really confirm that the whole is more than the sum of its parts. In fact, the information available from combined techniques goes beyond individual methods and offers a broader overview on the features of a given material.

In particular, recent works proposed: (*a*) a combination of X-ray and neutron diffraction for joint charge and spin density refinement; (*b*) calculations of variational wavefunctions constrained to fit experimental data, which enable the range of properties available from experimental density to be extended; (*c*) combination of X-ray scattering and NMR shielding.

It is clear that much work has still to be done to complete the framework combining all possible sources of information. Nevertheless, the results which have appeared in the last few years are extremely promising and certainly encourage further research.

## References

[bb1] Abramov, Yu. A. (1997). *Acta Cryst.* A**53**, 264–272.

[bb2] Arfken, G. (1985). *Mathematical Methods for Physicists.* Orlando, USA: Academic Press.

[bb3] Aronica, C., Jeanneau, E., El Moll, H., Luneau, D., Gillon, B., Goujon, A., Cousson, A., Carvajal, M. A. & Robert, V. (2007). *Chem. Eur. J.* **13**, 3666–3674. 10.1002/chem.200601253 17285651

[bb4] Ayers, P. W., Boyd, R. J., Bultinck, P., Caffarel, M., Carbó-Dorca, R., Causá, M., Cioslowski, J., Contreras-Garcia, J., Cooper, D. L., Coppens, P., Gatti, C., Grabowsky, S., Lazzeretti, P., Macchi, P., Martín Pendás, A., Popelier, P. L. A., Ruedenberg, K., Rzepa, H., Savin, A., Sax, A., Schwarz, W. H. E., Shahbazian, S., Silvi, B., Solà, M. & Tsirelson, V. (2015). *Comput. Theor. Chem.* **1053**, 2–16.

[bb5] Bader, R. F. W. (1990). *Atoms in Molecules: A Quantum Theory.* Oxford: Clarendon Press.

[bb6] Bader, R. F. W. & Gatti, C. (1998). *Chem. Phys. Lett.* **287**, 233–238.

[bb8] Bentley, J. & Stewart, R. F. (1973). *J. Comput. Phys.* **11**, 127–145.

[bb9] Bentley, J. & Stewart, R. F. (1974). *Acta Cryst.* A**30**, 60–67.

[bb10] Bibila Mayaya Bisseyou, Y., Bouhmaida, N., Guillot, B., Lecomte, C., Lugan, N., Ghermani, N. & Jelsch, C. (2012). *Acta Cryst.* B**68**, 646–660. 10.1107/S0108768112042826 23165601

[bb11] Bindzus, N., Straasø, T., Wahlberg, N., Becker, J., Bjerg, L., Lock, N., Dippel, A.-C. & Iversen, B. B. (2014). *Acta Cryst.* A**70**, 39–48. 10.1107/S2053273313026600 24419169

[bb12] Bonhomme, C., Gervais, C., Babonneau, F., Coelho, C., Pourpoint, F., Azaïs, T., Ashbrook, S. E., Griffin, J. M., Yates, J. R., Mauri, F. & Pickard, C. J. (2012). *Chem. Rev.* **112**, 5733–5779. 10.1021/cr300108a 23113537

[bb13] Boucherle, J. X., Gillon, B., Maruani, J. & Schweizer, J. (1987). *Mol. Phys.* **60**, 1121–1142.

[bb14] Bouhmaida, N., Ghermani, N.-E., Lecomte, C. & Thalal, A. (1997). *Acta Cryst.* A**53**, 556–563. 10.1107/s0108767399000689 10927284

[bb15] Brezgunova, M. E., Aubert, E., Dahaoui, S., Fertey, P., Lebègue, S., Jelsch, C., Ángyán, J. G. & Espinosa, E. (2012). *Cryst. Growth Des.* **12**, 5373–5386.

[bb16] Brown, P. J., Capiomont, A., Gillon, B. & Schweizer, J. (1979). *J. Magn. Magn. Mater.* **14**, 289–294.

[bb17] Bui, T. T. T., Dahaoui, S., Lecomte, C., Desiraju, G. R. & Espinosa, E. (2009). *Angew. Chem. Int. Ed.* **48**, 3838–3841. 10.1002/anie.200805739 19373823

[bb18] Capelli, S. C., Bürgi, H.-B., Dittrich, B., Grabowsky, S. & Jayatilaka, D. (2014). *IUCrJ*, **1**, 361–379. 10.1107/S2052252514014845 PMC4174878 25295177

[bb19] Claiser, N., Deutsch, M., Gillon, B., Gillet, J.-M., Lecomte, C., Luneau, D. & Souhassou, M. (2014). *Acta Cryst.* A**70**, C1083.

[bb20] Claiser, N., Souhassou, M., Lecomte, C., Gillon, B., Carbonera, C., Caneschi, A., Dei, A., Gatteschi, D., Bencini, A., Pontillon, Y. & Lelièvre-Berna, E. (2005). *J. Phys. Chem. B*, **109**, 2723–2732. 10.1021/jp0467907 16851280

[bb21] Cooper, M. J., Mijnarends, P., Shiotani, N., Sakai, N. & Bansil, A. (2004). *X-ray Compton Scattering.* Oxford University Press.

[bb22] Coulson, C. A. (1960). *Rev. Mod. Phys.* **32**, 170–177.

[bb23] Deutsch, M., Claiser, N., Pillet, S., Chumakov, Y., Becker, P., Gillet, J.-M., Gillon, B., Lecomte, C. & Souhassou, M. (2012). *Acta Cryst.* A**68**, 675–686. 10.1107/S0108767312031996 23075610

[bb24] Deutsch, M., Gillon, B., Claiser, N., Gillet, J.-M., Lecomte, C. & Souhassou, M. (2014). *IUCrJ*, **1**, 194–199. 10.1107/S2052252514007283 PMC4086435 25075338

[bb25] Domagała, S., Munshi, P., Ahmed, M., Guillot, B. & Jelsch, C. (2011). *Acta Cryst.* B**67**, 63–78. 10.1107/S0108768110041996 21245542

[bb26] Dos Santos, L. H. R., Genoni, A. & Macchi, P. (2014). *Acta Cryst.* A**70**, 532–551.

[bb27] Erba, A. & Pisani, C. (2012). *J. Comput. Chem.* **33**, 822–831. 10.1002/jcc.22907 22278778

[bb28] Espinosa, E., Molins, E. & Lecomte, C. (1998). *Chem. Phys. Lett.* **285**, 170–173.

[bb29] Farrugia, L. J. & Macchi, P. (2009). *J. Phys. Chem. A*, **113**, 10058–10067. 10.1021/jp9036588 19705813

[bb30] Fischer, A., Tiana, D., Scherer, W., Batke, K., Eickerling, G., Svendsen, H., Bindzus, N. & Iversen, B. B. (2011). *J. Phys. Chem. A*, **115**, 13061–13071. 10.1021/jp2050405 21863852

[bb31] Fournier, B., Bendeif, E., Guillot, B., Podjarny, A., Lecomte, C. & Jelsch, C. (2009). *J. Am. Chem. Soc.* **131**, 10929–10941. 10.1021/ja8095015 19594152

[bb32] Gadre, S. R. & Shrivastava, I. H. (1991). *J. Chem. Phys.* **94**, 4384–4391.

[bb33] Gatti, C. (2012). *Struct. Bond.* **147**, 193–286.

[bb34] Gatti, C. & Macchi, P. (2012). *Modern Charge Density Analysis.* New York: Springer.

[bb35] Gatti, C., Orlando, A. & Lo Presti, L. (2014). *Acta Cryst.* A**70**, C281.

[bb36] Genoni, A. (2013*a*). *J. Phys. Chem. Lett.* **4**, 1093–1099. 10.1021/jz400257n 26282026

[bb37] Genoni, A. (2013*b*). *J. Chem. Theory Comput.* **9**, 3004–3019. 10.1021/ct400293m 26583982

[bb38] Genoni, A. (2014). *Acta Cryst.* A**70**, C284.

[bb39] Ghermani, N. E., Bouhmaida, N. & Lecomte, C. (1992). *ELECTROS: Computer Program to Calculate Electroststic Properties from High Resolution X-ray Diffraction.* Internal Report URA CNRS 809, Université de Nancy I, France.

[bb40] Ghermani, N., Lecomte, C. & Bouhmaida, N. (1993). *Z. Naturforsch. Teil A*, **48**, 91–98.

[bb41] Gillet, J.-M. (2007). *Acta Cryst.* A**63**, 234–238. 10.1107/S0108767307001663 17435287

[bb96] Guillot, B., Enrique, E., Huder, L. & Jelsch, C. (2014). *Acta Cryst.* A**70**, C279.

[bb42] Haag, M. P., Vaucher, A. C., Bosson, M., Redon, S. & Reiher, M. (2014). *ChemPhysChem*, **15**, 3301–3319. 10.1002/cphc.201402342 25205397

[bb43] Hansen, N. K. & Coppens, P. (1978). *Acta Cryst.* A**34**, 909–921.

[bb44] Hayashi, H., Udagawa, Y., Gillet, J.-M., Calliebe, W. A. & Kao, C.-C. (2002). *Chemical Applications of Synchrotron Radiation*, edited by T. K. Sham, Vol. 12, *Advanced Series in Physical Chemistry.* Singapore: World Scientific.

[bb45] Herbst-Irmer, R. (2014). *Acta Cryst.* A**70**, C282.

[bb46] Hirshfeld, F. L. (1976). *Acta Cryst.* A**32**, 239–244.

[bb47] Hirshfeld, F. L. (1977). *Theor. Chim. Acta*, **44**, 129–138.

[bb48] Holladay, A., Leung, P. & Coppens, P. (1983). *Acta Cryst.* A**39**, 377–387.

[bb49] Iversen, B. B., Larsen, F. K., Figgis, B. N. & Reynolds, P. A. (1997). *J. Chem. Soc. Dalton Trans.* pp. 2227–2240.

[bb50] Jayatilaka, D. (1998). *Phys. Rev. Lett.* **80**, 798–801.

[bb51] Jayatilaka, D. (2012). *Modern Charge-Density Analysis*, edited by C. Gatti & P. Macchi, pp. 213–257. New York: Springer.

[bb52] Jayatilaka, D. & Dittrich, B. (2008). *Acta Cryst.* A**64**, 383–393. 10.1107/S0108767308005709 18421128

[bb53] Jayatilaka, D. & Grimwood, D. J. (2001). *Acta Cryst.* A**57**, 76–86. 10.1107/s0108767300013155 11124506

[bb54] Jelsch, C., Guillot, B., Lagoutte, A. & Lecomte, C. (2005). *J. Appl. Cryst.* **38**, 38–54.

[bb55] Jelsch, C., Domagala, S., Guillot, B., Liebschner, D., Fournier, B., Pichon-Pesme, V. & Lecomte, C. (2011). *Modern Charge Density Analysis*, edited by C. Gatti & P. Macchi. New York: Springer.

[bb56] Johnson, E., Keinan, S., Mori-Sánchez, P., Contreras-García, J., Cohen, A. & Yang, W. (2010). *J. Am. Chem. Soc.* **132**, 6498–6506. 10.1021/ja100936w PMC2864795 20394428

[bb57] Jørgensen, M. R. V., Hathwar, V. R., Bindzus, N., Wahlberg, N., Chen, Y.-S., Overgaard, J. & Iversen, B. B. (2014). *IUCrJ*, **1**, 267–280. 10.1107/S2052252514018570 PMC4174870 25295169

[bb58] Krause, L., Herbst-Irmer, R., Sheldrick, G. M. & Stalke, D. (2015). *J. Appl. Cryst.* **48**, 3–10. 10.1107/S1600576714022985 PMC4453166 26089746

[bb59] Kurki-Suonio, K. (1968). *Acta Cryst.* A**24**, 379–390.

[bb60] Li, X., Wu, G., Abramov, Y. A., Volkov, A. V. & Coppens, P. (2002). *Proc. Natl Acad. Sci. USA*, **99**, 12132–12137. 10.1073/pnas.192438999 PMC129410 12221293

[bb61] Macchi, P. (2013). *Crystallogr. Rev.* **19**, 58–101.

[bb62] Macchi, P., Bürgi, H.-B., Chimpri, A. S., Hauser, J. & Gál, Z. (2011). *J. Appl. Cryst.* **44**, 763–771.

[bb63] Martineau, C. (2014). *Solid State Nucl. Magn. Reson.* **63–64**, 1–12. 10.1016/j.ssnmr.2014.07.001 25112798

[bb64] Maslen, E. N., Fox, A. G. & O’Keefe, M. A. (1992). *International Tables for Crystallography*, Vol. C, edited by A. J. C. Wilson, pp. 476–511. Dordrecht: Kluwer Academic Publishers.

[bb65] Muzet, N., Guillot, B., Jelsch, C., Howard, J. A. K. & Lecomte, C. (2003). *Proc. Natl. Acad. Sci.* **100**, 8742–8747. 10.1073/pnas.1432955100 PMC166383 12855766

[bb66] Pavan, M. S., Durga Prasad, K. & Guru Row, T. N. (2013). *Chem. Commun.* **49**, 7558–7560. 10.1039/c3cc43513j 23872810

[bb67] Petricek, V., Dusek, M. & Palatinus, L. (2014). *Z. Kristallogr.* **229**, 345–352.

[bb68] Politzer, P. & Truhlar, D. (1981). *Chemical Applications of Atomic and Molecular Electrostatic Potentials.* New York: Plenum Press.

[bb69] Ressouche, E. (1999). *Physica B*, **267–268**, 27–36.

[bb70] Ressouche, E., Boucherle, J.-X., Gillon, B., Rey, P. & Schweizer, J. (1993). *J. Am. Chem. Soc.* **115**, 3610–3617.

[bb71] Sakakura, T., Nakano, T., Kimura, H., Noda, Y., Ishikawa, Y., Takenaka, Y., Tanaka, K., Kishimoto, S., Tokura, Y. & Miyasaka, S. (2014). *Acta Cryst.* A**70**, C280.

[bb72] Sakurai, Y., Itou, M., Barbiellini, B., Mijnarends, P. E., Markiewicz, R. S., Kaprzyk, S., Gillet, J.-M., Wakimoto, S., Fujita, M., Basak, S., Wang, Y. J., Al-Sawai, W., Lin, H., Bansil, A. & Yamada, K. (2011). *Science*, **332**, 698–702. 10.1126/science.1199391 21527674

[bb73] Saleh, G., Gatti, C. & Lo Presti, L. (2015). *Comput. Theor. Chem.* **1053**, 53–59.

[bb74] Saleh, G., Lo Presti, L., Gatti, C. & Ceresoli, D. (2013). *J. Appl. Cryst.* **46**, 1513–1517.

[bb75] Schmider, H., Edgecombe, K., Smith, V. H. & Weyrich, W. (1992). *J. Chem. Phys.* **96**, 8411–8419.

[bb76] Schmider, H., Smith, V. H. & Weyrich, W. (1993). *Z. Naturforsch. A*, **48**, 211–220.

[bb77] Schulz, T., Meindl, K., Leusser, D., Stern, D., Graf, J., Michaelsen, C., Ruf, M., Sheldrick, G. M. & Stalke, D. (2009). *J. Appl. Cryst.* **42**, 885–891.

[bb78] Schweizer, J. (2006). *Neutron Scattering from Magnetic Materials*, edited by T. Chatterji, ch. 4. Amsterdam: Elsevier.

[bb79] Shishkina, A. V., Zhurov, V. V., Stash, A. I., Vener, M. V., Pinkerton, A. A. & Tsirelson, V. G. (2013). *Cryst. Growth Des.* **13**, 816–828.

[bb80] Stewart, R. F. (1969). *J. Chem. Phys.* **51**, 4569–4578.

[bb81] Stewart, R. F. (1976). *Acta Cryst.* A**32**, 565–574.

[bb82] Stewart, R. F., Bentley, J. & Goodman, B. (1975). *J. Chem. Phys.* **63**, 3786–3793.

[bb83] Stewart, R. F. & Craven, B. M. (1993). *Biophys. J.* **65**, 998–1005. 10.1016/S0006-3495(93)81142-1 PMC1225816 8241415

[bb95] Stewart, R. F., Spackman, M. A. & Flensburg, C. (2000). *VALRAY User’s Manual*, Version 2.1. Carnegie-Mellon University, Pittsburg, USA, and University of Copenhagen, Denmark.

[bb84] Stokkebro Schmøkel, M., Bjerg, L., Overgaard, J., Krebs Larsen, F., Hellerup Madsen, G. K., Sugimoto, K., Takata, M. & Brummerstedt Iversen, B. (2013). *Angew. Chem. Int. Ed.* **52**, 1503–1506. 10.1002/anie.201206065 23239535

[bb85] Stoll, H., Wagenblast, G. & Preuβ, H. (1980). *Theor. Chim. Acta*, **57**, 169–178.

[bb86] Su, Z. & Coppens, P. (1992). *Acta Cryst.* A**48**, 188–197. 10.1107/s0108767391009820 1575938

[bb87] Taulelle, F. (2009). *Fundamental Principles of NMR Crystallography*, pp. 245–262. New York: John Wiley and Sons Ltd.

[bb88] Taulelle, F., Bouchevreau, B. & Martineau, C. (2013). *CrystEngComm*, **15**, 8613–8622.

[bb89] Volkov, A., Abramov, Y. A. & Coppens, P. (2001). *Acta Cryst.* A**57**, 272–282. 10.1107/s0108767300018547 11326112

[bb90] Volkov, A., King, H. F., Coppens, P. & Farrugia, L. J. (2006). *Acta Cryst.* A**62**, 400–408. 10.1107/S0108767306026298 16926487

[bb91] Volkov, A. V., Macchi, P., Farrugia, L. J., Gatti, C., Mallinson, P., Richter, T. & Koritsanszky, T. (2006). *XD2006.* University at Buffalo, State University of New York, NY, USA; University of Milano, Italy; University of Glasgow, UK; CNRISTM, Milano, Italy; Middle Tennessee State University, TN, USA.

[bb92] Wahlberg, N., Bindzus, N., Bjerg, L., Becker, J. & Iversen, B. (2014). *Acta Cryst.* A**70**, C283.

[bb93] Wenger, E., Dahaoui, S., Alle, P., Parois, P., Palin, C., Lecomte, C. & Schaniel, D. (2014). *Acta Cryst.* B**70**, 783–791. 10.1107/S2052520614017338 25274511

[bb94] Weyrich, W. (1996). *Quantum Mechanical Ab-initio Calculation of the Properties of Crystalline Materials*, edited by D. C. Pisani. pp. 245–272. New York: Springer.

